# Factors associated with dietary patterns (DPS) and nutritional status among pregnant women in AM-HDSS, South Ethiopia

**DOI:** 10.3389/fnut.2024.1443227

**Published:** 2024-09-12

**Authors:** Teshale Fikadu, Dessalegn Tamiru, Beyene Wondafrash Ademe

**Affiliations:** ^1^School of Public Health, College of Medicine and Health Science, Arba Minch University, Arba Minch, Ethiopia; ^2^Department of Nutrition and Dietetics, Institute of Health, Jimma University, Jimma, Ethiopia

**Keywords:** associated factor, dietary patterns, dietary practice, nutritional status, pregnant women

## Abstract

**Background:**

Malnutrition during pregnancy increases the risk of chronic illness later in life and adverse birth outcomes in subsequent generations. In this regard, consumption of diets rich in adequate energy, protein, vitamins, and minerals from a variety of foods is essential. Evidence on the status of maternal dietary pattern is very crucial. Hence, the aim of this study was to assess factors associated with dietary patterns and nutritional status of pregnant women in South Ethiopia.

**Methods:**

A community-based cross-sectional study was conducted among 638 randomly selected pregnant women using a validated, a pre-tested, contextualized food frequency questionnaire using interviewer-administered structured questionnaire by digital open-source toolkit. Principal component factor analysis was employed to determine dietary patterns. Bivariable and multivariable ordinal logistic regression analyses were used to identify factors associated with dietary patterns and nutritional status, using STATA version 16.

**Result:**

The dietary habits of pregnant women were best explained by three distinct dietary patterns. Urban dwellers (AOR = 2.18; 95% CI: 1.33, 3.59), from high socio-economic status (AOR = 2.43; 95% CI: 1.68, 3.51), from middle socio-economic status (AOR = 1.72; 95% CI: 1.19, 2.48), primigravida mothers (AOR = 1.72; 95% CI: 1.07, 2.78), and multigravida mothers (AOR = 2.08; 95% CI: 1.39, 3.10) were high likelihood to consume the highest tercile of “Cereals-Pulses and Dairy” compared to rural dwellers, from low socio-economic status and grand multigravida, respectively. Attending formal education (AOR = 1.60; 95% CI: 1.02, 2.51), from higher socioeconomic status (AOR = 1.56; 95% CI: 1.02, 2.38), not having food aversion (AOR = 1.98; 95% CI: 1.16, 3.39), and had good dietary knowledge (AOR = 2.16; 95% CI: 1.08, 4.32) were associated with a higher tercile consumption of “Nutrient-Dense” food compared to those without formal education, having food aversion and had poor dietary knowledge, respectively. Not attending formal education (AOR = 2.22; 95% CI: 1.48, 3.36), had decision-making autonomy (AOR = 1.91; 95% CI: 1.26, 2.90), and had good dietary knowledge (AOR = 1.86; 95% CI: 1.13, 3.08) were found to consume the highest tercile of “Leafy local food” compared to their counterpart. Consumption of lower terciles “Nutrient-Dense” food (AOR = 1.63; 95% CI: 1.07, 2.47) and “Leafy local food” (AOR = 2.32; 95% CI: 1.54, 3.51) were found to be factors associated with under nutrition during pregnancy.

**Conclusion:**

Three distinct dietary patterns were identified. Factors associated with these major dietary patterns included place of residence, socio-economic status, educational level, dietary knowledge, food aversion, number of pregnancies, and maternal decision-making autonomy. Under nutrition among pregnant women was found to be high and associated with the consumption of ‘Nutrient-Dense’ and ‘Leafy local’ foods. Therefore, concerned health authorities should strengthen dietary counseling during pregnancy, provide family planning services, and promote women’s education.

## 1 Introduction

Pregnancy is a critical time in which the health and well-being of the next generation can be determined. Through the concept of ‘nutrition programming’ malnutrition during pregnancy can lead to long-term effects such as an increased risk for chronic illness later in life, and adverse birth outcomes within the subsequent generation (1, 2).

Pregnant women are recommended to consume diets rich in adequate energy, protein, vitamins, and minerals from a variety of foods, maintain a healthy lifestyle, monitor weight gain during pregnancy, participate in regular exercise, and avoid smoking and alcohol (3–5). Nonetheless, many pregnant women consume insufficient amounts of fruits, vegetables, meat, and dairy products to meet their nutritional needs due to a lack of dietary diversity, which can result in malnutrition, (6, 7) particularly micronutrient deficiencies (4). Understanding the dietary pattern and implementing socioculturally customized interventions are essential to address the gap (8).

Dietary practices are closely linked to the socio-economical, socio-cultural, anthropological, and psychological aspects of an individual’s environment. As such, accepting both the dietary patterns and the characteristics of the groups that most commonly practice them is crucial. This knowledge is a vital step toward developing tailored interventions that address the specific needs of each group effectively (8, 9). This identification appears to be a suitable alternative for evaluating dietary exposures in nutritional epidemiology (10).

The dietary pattern analysis approach provides a more holistic understanding of dietary habits and helps explain how socioeconomic factors influence adherence to these patterns, which encompass combinations of commonly consumed foods (11). This approach helps researchers and policymakers focus on tailored interventions to address nutrition-related health problems, as it provides a complementary method for comprehending individual’s eating habits. Instead of concentrating solely on specific nutrients, it considers the variety of foods consumed and capturing how they interact within a diet (12, 13). This broader viewpoint is essential for understanding the intricacies of human nutrition and its effects on health. Therefore, dietary pattern analysis is vital for gaining insights into how diet impacts the health of both mothers and their offspring during pregnancy (12, 13). It is typically relies on data-driven approach, where patterns derived through statistical modeling of real-world dietary data. This approach offers valuable insights into dietary behaviors by reflecting actual consumption patterns and offering additional information about overall diet quality (1, 13).

Studies indicate that dietary pattern during pregnancy impact their weight gain, which inextricably linked into the nutritional status of the mother (5, 14). Also marital status, income, educational status, religion, occupation and number of delivery were associated with dietary pattern during pregnancy (15, 16). In addition recent study conducted in Ethiopia shows that the consumption of eggs, dairy products, pulses, vegetables, and fruits during pregnancy is associated with increased birth weights (17). Despite this, nearly half of the pregnant women in the area suffer from under nutrition (17). This highlights the importance of considering dietary patterns rather than focusing on specific food items. And improving the nutritional status of pregnant women through the promotion of dietary patterns, rather than a single-nutrient approach, provides greater overall benefits (18).

Despite various interventions in Ethiopia, including dietary counseling on healthy eating, counseling on appropriate weight gain during pregnancy, and iron-folic acid supplementation (4, 19), as well as the development and implementation of food-based dietary guidelines to alleviate malnutrition (20), under nutrition during pregnancy remains a public health problem, with a pooled prevalence of 32% (6). Factors associated with nutrition during pregnancy include residence, maternal education, and age at first marriage, number of Antenatal care (ANC) visits, substance use, prenatal dietary advice, intestinal parasite infection, meal skipping, household food insecurity, and dietary diversity (6, 21–23). However, evidence on factors associated with dietary patterns and their effects on nutritional status among pregnant women is scarce. Thus, this study aims to identify factors associated with major dietary patterns and their effects on maternal nutritional status during pregnancy in Arba Minch Health and Demographic Surveillance site (AM-HDSS) Gamo Zone, South Ethiopia.

## 2 Materials and methods

### 2.1 Study setting, design, and period

A community-based cross-sectional study was employed between October 1*^st^*, 2023 and March 1*^st^*, 2024, among 638 randomly selected pregnant women, at their 16 to 20 weeks of gestation in the (AM-HDSS). The data served as a baseline for a prospective cohort study aimed to assess the effect of dietary practices on the nutritional status of newborns. AM-HDSS is located in the Arba Minch Zurea and Gacho Baba districts, found approximately 437 km and 479 km, respectively, to the south of Addis Ababa, the capital city of Ethiopia. The site encompasses nine kebeles, six of which are found in the Arba Minch district and three in the Gacho Baba district. In the surveillance site, 2,598 women were expected to be pregnant for the year 2023/24 and receive healthcare services at 7 nearby health centers, 37 health posts, and private healthcare facilities. The dietary practices of the population primarily consist of starchy staples, as the commonly grown crops include maize, barley, and tubers such as potatoes, sweet potatoes, and taro. Additionally, the area produces a surplus of fruits, vegetables, and moringa stenopetala.

### 2.2 Source population and study population

All pregnant women found in AM-HDSS were the source population, whereas the study populations were all selected pregnant women fulfilled the inclusion criteria found in AM-HDSS.

### 2.3 Inclusion and exclusion criteria

All permanent residents of AM-HDSS pregnant women were the source population, and all selected pregnant women were included in the study. However, critically ill, those who did not volunteer to participate, and women with known chronic illnesses such as HIV and diabetes mellitus were excluded.

### 2.4 Sample size determination and sampling procedure

The sample size was calculated using the single population proportion formula, with parameters set at a 95% confidence interval, a 5% degree of precision, and an anticipated prevalence of under nutrition among pregnant women at 34.36%, as reported in a systematic review and meta-analysis (6).

$$\text{n}=\frac{{\left({\text{Z}}_{1 -\alpha /2}\right)}^{2}*P\,\left(1 -P\right)}{{\left(\text{d}\right)}^{2}}$$

Where

*n* = Desired number of samples

*P* = Expected Proportion of under nutrition among pregnant women = 34.36%

*Z*_1–a/2_ = Standard value for 95% CI = 1.96

The calculated sample size, adjusted for a potential 10% non-response rate, was 381. However, a total of 638 pregnant women were evaluated at baseline to determine the exposure status of participants for the cohort study. Therefore, all 638 pregnant women were included in this study. The sample was allocated proportionally to each kebele based on the expected number of pregnant women, and a computer-generated simple random sampling method was employed to recruit the study participants.

### 2.5 Data collection procedures

Data were obtained through a pre-tested, interviewer-administered, structured questionnaire developed and used to collect data on socio-demographic, health-related, and dietary factors. Dietary intake was assessed using a modified and validated food frequency questionnaire (FFQ) administered over the previous week. This FFQ included 46 food items tailored to the local context. Trained data collectors used the digital open-source toolkit Kobo Collect for data collection. A pre-designed questionnaire template was deployed to the server and downloaded onto the data collectors’ cell phones. Subsequently, the collected data were uploaded to the server. A one-week dietary history, consisting of 46 food items grouped into ten food categories, were collected to determine the dietary patterns of a pregnant women.

### 2.6 Operational definitions and measurement

Maternal dietary knowledge: Knowledge was assessed using questions developed from similar studies. Incorrect responses were coded as “0” indicating a lack of awareness, while correct responses were coded as “1” indicating awareness, with scores ranging from minimum 0% to maximum 100%. Based on Bloom’s cut-off point for knowledge, mothers who scored less than 60% were considered to have “poor knowledge,” those scoring between 60% and 79% were considered to have “moderate knowledge,” and those scoring 80% or higher were considered to have “good knowledge” (24).

Maternal nutritional status: Nutritional status was assessed using Mid-Upper Arm Circumference (MUAC), which measures fat-free mass and the circumference of the upper arm at the midpoint between the acromion processes and olecranon (7). Under nutrition in pregnant women that increases the risk of adverse pregnancy outcomes is defined as MUAC < 23 cm (25).

Household Wealth Status: The household wealth status was determined using items adapted from the 2016 Ethiopian Demographic and Health Survey (EDHS). These items included the number of livestock owned, the availability of agricultural land, the materials used in constructing the house (such as floor, walls, and roof), the source of drinking water, the presence of electricity, the type of cooking fuel used, and ownership of modern household goods and livestock (such as a bicycle, television, radio, motorcycle, sewing machine, telephone, car, refrigerator, mattress, bed, and mobile phone). The assumption was that possession of these assets, services, and amenities is indicative of the relative economic position of the household (26).

### 2.7 Data process and analysis

Data were reviewed online, edited, and verified daily for consistency and completeness. After data collection was completed, the data were downloaded in Excel format and exported to STATA version 16.0 for analysis. Principal-component factor (PCF) analysis was used to assess the socioeconomic status of households. Variables or assets that were owned by more than 95% or less than 5% of the participants were excluded, as they were not help to distinguish between richer and poorer. After initial exploration the variables with frequency analysis, we evaluated the assumptions for PCF using the Kaiser-Meyer-Olkin measure and Bartlett’s test of sphericity. Next, variables with cross-loadings or complex structures were removed. Finally, three components which explained a total variance of 67.7% were extracted and the first component explained the largest variance was used to categorize wealth status.

Likewise PCF analysis and reliability analysis were employed to determine the dimension and internal consistency of items used to construct a composite score for maternal dietary knowledge. The questions “Is it recommended to increase the amount of dietary intake during pregnancy?”, “Does inadequate diet during pregnancy lead to maternal under nutrition?”, and “Does inadequate diet during pregnancy lead to low birth weight?” were removed due to cross-loading or complex structures. The remaining 15 items, with satisfactory internal consistency (Cronbach’s alpha = 0.74), were used to score maternal dietary knowledge.

Dietary patterns were determined using an exploratory factor analysis (EFA) based on weekly dietary consumption frequency. To enhance comparability and minimize complexity of the factor analysis for identifying factors that explain the highest variation, individual food items consumed were categorized following existing literature and contextualized (27). All assumptions of exploratory factor analysis were reviewed, and variables that violated these assumptions were excluded. The adequacy of the sample-to-factors ratio was assessed using the Kaiser–Meyer–Olkin (KMO) (with a *p*-value > 0.05) and Bartlett’s test of sphericity (with a *p*-value < 0.05).

To identify interpretable and independent dietary patterns, orthogonal rotation and factor loadings above 0.5 were employed in the final model. Unbiased and robust estimates of the true factor scores, based on the factor loadings of consumption frequency, were generated using the Bartlett procedure. The factor scores were then ranked into three terciles (low, medium, and high) and considered as outcome variables. Lower factor scores typically indicate less frequent dietary consumption (28).

Factor loadings for each food item were computed, and the resulting factors were identified as distinct dietary patterns (15). Experts and authors then meticulously reviewed the food items within each factors “distinct dietary pattern” and assigned a name that best described and included all the items.

Ordinal logistic regression (OLS) analysis was conducted. All explanatory variables with an association to each dietary pattern tercile at a p-value less than 0.25 in the bivariable analysis were included in the multivariable model. And a multivariable OLS analysis was performed to determine factors associated with each dietary pattern. Odds ratios with 95% confidence intervals were used to decide statistical significance. The proportional odds assumption and constant slope parameter across categories were assessed through the Brant test of parallel lines. Model fitness was evaluated using the Ordinal Hosmer and Lemeshow goodness-of-fit test, PR(deviance) test, and Lipsitz test, with a minimum *p*-value of 0.68 for all tests applied to the three models. Multicollinearity among independent variables was examined using variance inflation factor (VIF) the maximum VIF was 1.65.

### 2.8 Ethical consideration

The study conducted in line with the principles of the Declaration of Helsinki (29). Approval and clearance for ethics was obtained from the Institutional Research Ethics Review Board (IRB) of Institute of Health, Jimma University under letter reference number (Nut/5029/2023). Cooperation letter was obtained from the Gamo Zone Health Office, and formal permissions were obtained from local administrators. Pregnant women were linked with healthcare facilities for antenatal care (ANC), where they received dietary counseling and nutritional education based on the WHO 2016 ANC guideline. Informed consent was obtained from the participants, and to maintain confidentiality, their personal details were not recorded in the survey tool.

## 3 Result

### 3.1 Background characteristics of pregnant women in AM-HDSS, South Ethiopia

The study included 638 pregnant women at 16 to 20 weeks of gestation, and with a mean age of 26 ± 5 years. About 90.28% and 62.85% of the participants were rural residents and attended formal education, respectively. Majority of the participants, 67.08%, were housewives. Of the participants, 33.23% were from high-wealth status, 32.13% did not receive antenatal care (ANC), and 38.71% had not started taking iron-folic acid supplements yet. Only 16.61% of participants received information about dietary intake during pregnancy, whereas 10.97% had good dietary knowledge (Table 1).

**TABLE 1:** Background characteristics including nutritional status of pregnant women in AM-HDSS, South Ethiopia.

Variable	Category	Participant No (%)	MUAC		*P*-value
			<23 cm	>23 cm	
Age of the respondent	Less than 20 years	81 (12.70)	25 (12.25)	56 (12.90)	0.94
	20 to 35 years	514 (80.56)	166 (81.37)	348 (80.18)	
	36 years and more	43 (6.74)	13 (6.37)	30 (6.91)	
Place of residence	Rural	576 (90.28)	184 (90.20)	392 (90.32)	0.96
	Urban	62 (9.72)	20 (9.80)	42 (9.68)	
Religion	Orthodox	123 (19.28)	41 (20.10)	82 (18.89)	0.72
	Protestant	515 (80.72)	163 (79.90)	352 (81.11)	
Educational status	No formal education	237 (37.15)	81 (39.71)	156 (35.94)	0.36
	Formal education	401 (62.85)	123 (60.29)	278 (64.06)	
Occupational status	House wife	428 (67.08)	138 (67.65)	290 (66.82)	0.01
	Farmer	82 (12.85)	21 (10.29)	61 (14.06)	
	Merchant	74 (11.60)	35 (17.16)	39 (8.99)	
	Government employ	34 (5.33)	7 (3.43)	27 (6.22)	
	Student	20 (3.13)	3 (1.47)	17 (3.92)	
Households decision-making	By both	118 (18.50)	26 (12.75)	92 (21.20)	0.01
	Mainly by husband	520 (81.50)	178 (87.25)	342 (78.80)	
Household wealth status	Low	218 (34.17)	58 (28.43)	160 (36.87)	0.11
	Middle	208 (32.60)	72 (35.29)	136 (31.34)	
	High	212 (33.23)	74 (36.27)	138 (31.80)	
Nutritional knowledge	Had poor knowledge	298 (46.71)	96 (47.06)	202 (46.54)	0.72
	Had moderate knowledge	270 (42.32)	83 (40.69)	187 (43.09)	
	Had good knowledge	70 (10.97)	25 (12.25)	45 (10.37)	
Number of pregnancy	Primigravida	141 (22.10)	39 (19.12)	102 (23.50)	0.04
	Multigravida	376 (58.93)	115 (56.37)	261 (60.14)	
	Grand Multi	121 (18.97)	50 (24.51)	71 (16.36)	
Number of delivery	Primipara	154 (31.49)	51 (32.08)	103 (31.21)	0.98
	Multipara	276 (56.44)	89 (55.97)	187 (56.67)	
	Grand Multipara	59 (12.07)	19 (11.95)	40 (12.12)	
ANC visit	Yes	433 (67.87)	132 (64.71)	301 (69.35)	0.24
	No	205 (32.13)	72 (35.29)	133 (30.65)	
Had nausea/vomiting	Often	135 (21.16)	58 (28.43)	77 (17.74)	0.01
	Some times	204 (31.97)	50 (24.51)	154 (35.48)	
	No	299 (46.87)	96 (47.06)	203 (46.77)	
Food aversion	Yes	91 (14.26)	24 (11.76)	67 (15.44)	0.21
	No	547 (85.74)	180 (88.24)	367 (84.56)	
Nutritional counseling	Yes	106 (16.61)	31 (15.20)	75 (17.28)	0.51
	No	532 (83.39)	173 (84.80)	359 (82.72)	
Iron/folic acid supplementation	Yes	391 (61.29)	116 (56.86)	275 (63.36)	0.12
	No	247 (38.71)	88 (43.14)	159 (36.64)	

### 3.2 Major dietary patterns of pregnant women in AM-HDSS, South Ethiopia

The dietary habits of pregnant women were best explained by three factors (distinct dietary patterns), which explain 60% of the total variation. The first factor, which explains 25.63% of the total variance, includes foods such as tubers (Potato, Sweet Potato, Taro, and Beetroot), vegetables (Collard Greens, Onion, Garlic, and Green Pepper), fruits (Banana, Mango, Avocado, and Papaya), meat (Raw Meat, Organ Meat, Fish, and Chicken), and eggs. Since this distinct dietary pattern encompasses the intake of carbohydrates, proteins, fats, and micronutrients, it is labeled as “Nutrient-Dense”.

The second factor, which explains 18.58% of the total variance, includes foods such as cereals (Teff, Maize, Barley, Wheat, Sorghum, and Rice), dairy products (Milk and milk products), and legumes (Pea, Bean, Lentil, and Chickpea). This distinct dietary pattern is labeled as “Cereals-Pulses and Dairy”. And the third factor, which explains 15.92% of the total variance, includes foods such as Moringa stenopetala leaves in various forms and a locally prepared coffee leaf tea beverage made with ginger, garlic, rosemary, African wormwood, anise, Artemisia abyssinica, thyme, coriander, and roasted coffee leaves. Since this distinct dietary pattern consists of locally consumed food and beverages made from leaves, it is labeled as “Leafy Local Food” (Table 2).

**TABLE 2:** Summary of the major dietary patterns and the contribution of each food group to the total variance explained by these dietary patterns among pregnant women in AM-HDSS, South Ethiopia.

	Food groups	Factor 1^ß^	Factor 2^ß^	Factor 3^ß^
1	Cereal		0.652	
2	Dairy		0.648	
3	Pulses		0.634	
4	Tubers	0.526		
5	Vegetables	0.535		
6	Fruits	0.595		
7	Meat	0.684		
8	Egg	0.747		
9	Leafy local food			0.891
Total variance (60.13%)		18.58%	25.63%	15.92%

^ß^ Factor 1^ß^ refers to “nutrient dense foods” Factor 2^ß^ refers to “Cereal-pulses and dairy”, and Factor 1^ß^ refers to “Leafy local food.”

### 3.3 Factors associated with major dietary pattern

Among all candidate variables (Supplementary Table 1) for multivariable analysis, seven were identified as factors associated with major dietary patterns. These include place of residence, educational status, household wealth status, maternal decision-making autonomy, maternal dietary knowledge, number of pregnancies, and food aversion.

Urban women were 2 times more likely to consume the highest tercile of “Cereals-Pulses and Dairy” compared to rural women (AOR = 2.18; 95% CI: 1.33, 3.59). Mothers with their first pregnancy (AOR = 1.72; 95% CI: 1.07, 2.78) and those with two to five pregnancies (AOR = 2.08; 95% CI: 1.39, 3.10) were 1.7 and 2 times more likely, respectively, to consume the highest tercile of “Cereals-Pulses and Dairy” compared to mothers with more than five pregnancies. The odds of consuming the highest tercile of “Cereals-Pulses and Dairy” were about 2.5 times and 1.7 times higher among women from high and middle socioeconomic status compared to those from low socioeconomic status respectively (AOR = 2.43; 95% CI: 1.68, 3.51) and (AOR = 1.72; 95% CI: 1.07, 2.78).

Women who attended formal education (AOR = 1.60; 95% CI: 1.02, 2.51) and came from higher socioeconomic statuses (AOR+ 1.56; 95% CI: 1.02, 2.38) were 1.6 times more likely consume higher tercile of “Nutrient-Dense” food compared to their counterparts. Also, the odds of consume the highest tercile of “Nutrient-Dense” foods were 2 times higher among women who do not avert food during pregnancy (AOR = 1.98; 95% CI: 1.16, 3.39) and among those with good dietary knowledge (AOR = 2.16; 95% CI: 1.08, 4.32).

Women who don’t attend formal education (AOR = 2.22; 95% CI: 1.48, 3.36), and had decisions making autonomy (AOR = 1.91; 95% CI: 1.26, 2.90) were 2 times more likely to consume higher tercile of “Leafy local food” compared to their counterparts. Also Participants with good dietary knowledge were 1.86 times more likely to consume the higher tercile of ‘Leafy Local Food’ compared to those with poor dietary knowledge (AOR = 1.86; 95% CI: 1.13, 3.08) (Table 3).

**TABLE 3:** Factors associated with major dietary pattern among pregnant women in AM-HDSS, South Ethiopia.

Variable	Category	COR(95% CI)	AOR(95% CI)
“Cereals-Pulses and Dairy”			
Place of residence	Rural	Ref	Ref
	Urban	2.45 (1.5, 3.96)	2.18 (1.33, 3.59)
Household wealth status	Low	Ref	Ref
	Middle	1.93 (1.36, 2.75)	1.72 (1.19, 2.48)
	High	2.50 (1.74, 3.59)	2.43 (1.68, 3.51)
Number of pregnancy	Primigravida	2.12 (1.34, 3.34)	1.72 (1.07, 2.78)
	Multigravida	2.14 (1.45, 3.15)	2.08 (1.39, 3.10)
	Grand Multigravida	Ref	Ref
“Nutrient-Dense”			
Educational status	No formal education	Ref	Ref
	Formal education	1.48 (1.10, 1.99)	1.60 (1.02, 2.51)
Household wealth status	Low	Ref	
	Middle	1.24 (0.88, 1.76)	1.51 (0.98, 2.32)
	High	1.47 (1.04, 2.08)	1.56 (1.02, 2.38)
Nutritional knowledge	Poor knowledge	Ref	Ref
	Moderate knowledge	0.90 (0.67, 1.22)	1.77 (0.96, 3.29)
	Good knowledge	1.43 (0.88, 2.31)	2.16 (1.08, 4.32)
Food aversion	Yes	Ref	Ref
	No	1.83 (1.20, 2.79)	1.98 (1.16, 3.39)
“Leafy local food”			
Educational status	No formal education	2.20 (1.63, 2.98)	2.22 (1.48, 3.36)
	Formal education	Ref	Ref
Decision-making autonomy	By both	1.52 (1.06, 2.19)	1.91 (1.26, 2.90)
	Mainly by husband	Ref	Ref
Household wealth status	Low	1.49 (1.05, 2.11)	1.60 (1.12, 2.30)
	Middle	1.33 (0.94, 1.90)	1.27 (0.88, 1.83)
	High	Ref	Ref
Nutritional knowledge	Poor knowledge	Ref	Ref
	Moderate knowledge	1.44 (1.06, 1.95)	1.52 (1.11, 2.09)
	Good knowledge	1.74 (1.07, 2.83)	1.86 (1.13, 3.08)

### 3.4 Under nutrition and its association with major dietary pattern among pregnant women in AM-HDSS, South Ethiopia

Two hundred and four 31.97% of the pregnant women were undernourished (MUAC < 23 cm). “Nutrient-Dense” and “Leafy local food” were significantly associated with under nutrition during pregnancy. The odds of under nutrition during pregnancy were 1.6 times higher among women who consumed the lower tercile of “Nutrient-Dense” food compared to those in the higher tercile (AOR = 1.63; 95% CI: 1.07, 2.47). Similarly, participants who consumed the lower tercile of “Leafy local food” were 2.3 times more likely to be under nourished compared to those consumed the higher tercile (AOR = 2.32; 95% CI: 1.54, 3.51) (Table 4).

**TABLE 4:** Under nutrition and its association with major dietary pattern among pregnant women in AM-HDSS, South Ethiopia.

Variable	Category	COR(95% CI)	AOR(95% CI)
“Cereals-Pulses and Dairy”	Low	1.23 (0.81, 1.83)	1.30 (0.86, 1.98)
	Medium	1.15 (0.76, 1.73)	1.23 (0.81, 1.88)
	High	Ref	Ref
“Nutrient-Dense”	Low	1.59 (1.06, 2.41)	1.63 (1.07, 2.47)
	Medium	1.19 (0.78, 1.81)	1.17 (0.77, 1.79)
	High	Ref	Ref
“Leafy local food”	Low	2.26 (1.49, 3.39)	2.32 (1.54, 3.51)
	Medium	0.99 (0.64, 1.53)	1.01 (0.65, 1.57)
	High	Ref	Ref

## 4 Discussion

Human dietary consumption is complex, making reports of habitual dietary consumption based on individual food intake unreliable. Therefore, a robust dietary analysis is a more feasible method for characterizing dietary habits, quality, and the consequences of habitual dietary practices (28). Thus this study aims to assess factors associated with DPS and nutritional status among pregnant women in South Ethiopia.

In the current study, three major distinct dietary patterns namely, “Cereals-Pulses and Dairy”, “Nutrient-Dense” and “Leafy local food” were identified. Similar previous studies have identified different dietary patterns among pregnant women, and these patterns were associated with adverse pregnancy outcomes such as gestational hypertension, preeclampsia, large for gestational age, gestational diabetes mellitus, perinatal depression, preterm birth, and low birth weight (30–34).

As the number of pregnancies increases, exposure to health facilities and health information, including healthy dietary practices, also increases, which consequently improves the dietary habits of pregnant women. Contrary to this, the current study reported that the odds of consuming a higher tercile of the “Cereals-Pulses and Dairy” dietary pattern were higher among primigravida and multigravida mothers compared to grand multigravida mothers. This is probably due to the increased number of family members among Multigravida mothers (35).

The probability of consuming higher tercile of the “Cereals-Pulses and Dairy” pattern and “Nutrient-Dense” pattern were higher among mothers from higher socioeconomic statuses compared to those from lower socioeconomic statuses. This can be attributed to the fact that the purchasing power of these mothers is relatively better within these groups, leading to an increased likelihood of consumption. Similarly, in Ethiopia, food price inflation has reached over 62%, making it non-affordable for most of the poor in the country, which hinders access to consumption (36, 37). Our finding was incongruent with the findings in eastern Ethiopia, where the odds of legume consumption among pregnant women were higher among mothers from low socioeconomic status (28). Another study from Nigeria also observed higher consumption of protein-rich foods and legumes among mothers with low income (15). Additionally, a study from Ethiopia indicated that per capita consumption of major cereals was higher among individuals from low socioeconomic status (38). These variations could be due to differences in geographical location, measurement techniques, food costs, and disparities in time and place.

The odds of consuming a higher tercile of the “Leafy local food” pattern were higher among mothers from lower socioeconomic statuses compared to those from higher socioeconomic statuses. This is likely due to the availability and low cost of fresh moringa stenopetala leaves and the ingredients used to prepare coffee leaf tea beverage. Additionally, more than 90% of the participants were from rural areas where these trees are cultivated, (39) which further increases the likelihood of consumption.

The likelihood of consuming the highest tercile of the “Cereals-Pulses and Dairy” pattern was higher among mothers living in urban areas than those living in rural areas. This is consistent with previous evidence, which showed that urban pregnant women had higher legume consumption. (28), and another study revealed that the major cereal per capita consumption of urban dwellers was 51%, compared to 46% among rural dwellers (38).

Good dietary knowledge and practices are indispensable during pregnancy; however, more than half of Ethiopian pregnant women have poor knowledge and dietary practices (40–42). This widespread lack of dietary knowledge, combined with low literacy levels and poor socioeconomic status, predisposes pregnant women to poor dietary habits and under nutrition consequently leads to adverse pregnancy outcome. A similar finding was reported in this study, showing that mothers with good dietary knowledge had higher odds of consuming the “Nutrient-Dense” pattern and the “Leafy Local Foods” pattern compared to those with poor dietary knowledge.

The likelihood of consuming a higher tercile of the “Nutrient-Dense” pattern was higher among women who attend formal education compared to those without formal education. This is supported by previous evidence showing that education helps individuals acquire knowledge about healthy and high-quality dietary practices.(43). Similarly, as the level of education increases, exposure to health information, utilization of maternal health services, and continuum of maternal care also increase. These factors lead to better dietary knowledge and practices, which consequently reduce under nutrition and adverse pregnancy outcomes (44, 45). But surprisingly, consuming a higher tercile of the “Leafy local food” pattern was higher among participants without formal education. This is probably due to local and cultural dietary habits are deeply ingrained among rural populations and individuals with low literacy levels (46).

The odds of consuming a higher tercile of the “Leafy Local Food” pattern were higher among women who had decision-making autonomy compared to their counterparts. This might be due to higher continuum of care utilization among pregnant women who have autonomy in healthcare decision-making (47) which in turn increases dietary knowledge and practices during pregnancy. In contrast, women’s decision-making autonomy in low- and middle-income countries, including Ethiopia, is limited, which affects health service utilization among women of reproductive age (48, 49). As a result, they often lack information about healthy dietary practices and are at risk of under nutrition (21).

Food aversion is common during pregnancy due to hormonal changes and primary physiological barriers to optimal dietary practices (50). This idea was supported by the current study, which found that the odds of consuming a higher tercile of the “Nutrient-Dense” pattern were higher among women without food aversion compared to their counterpart. This finding is congruent with a previous study, which reported that fruits and animal-source foods were consumed more frequently among pregnant women without food aversion (28).

The level of under nutrition among pregnant women in the study area was 31.97%. which is similar to the pooled prevalence in Ethiopia and South Ethiopia, (6) but lower than the pooled prevalence in Oromia regional state of Ethiopia and other pocket studies (6, 7, 21, 22, 51, 52). However, it was higher than the pooled prevalence in Amhara regional state of Ethiopia and other pocket studies (6, 23, 53). These differences were probably due to differences in dietary habits and cultural food taboos.

In the current study, the odds of under nutrition were 1.63 times higher among participants who consumed the lower tercile of the “Nutrient-Dense” pattern compared to those who consumed the higher tercile. This highlights that consuming a healthy and adequate diet during pregnancy promotes weight gain, reduces the risk of diseases, and improves nutritional status (54, 55). The finding of this study is congruent with previous evidence, which shows that a lack of meat and fruit consumption is associated with a higher incidence of under nutrition during pregnancy (56).

This study indicated that the odds of under nutrition were 2.32 times higher among women who consumed the lower tercile of the “Leafy Local Food” pattern compared to those who consumed the higher tercile. This is probably due to the nutrient-rich content of Moringa stenopetala leaf (which provide macro and micronutrients) (57, 58) and the antioxidant, anti-emetic., anti-inflammatory, antibacterial, and antifungal effects of coffee leaf tea. Both contribute to improved dietary intake and nutritional status (59, 60). Also the macro and micro nutrient content of the coffee leaf tea helps to improve maternal nutritional status (61).

### 4.1 Practical implication

Targeted nutrition programs should focus on increasing the intake of “Nutrient-Dense” pattern and “Leafy Local Food” pattern to prevent under nutrition.

Educating pregnant women about the importance of nutrition during pregnancy, including dietary diversity and specific dietary patterns, could improve maternal nutritional status and pregnancy outcomes. Additionally, programs could integrate nutrition education into formal schooling and community-based adult education. Enhancing dietary education and providing information about healthy dietary practices can be a key component of maternal health programs.

Tailored interventions might be needed for rural women to improve their dietary practice and under nutrition. Local food practices should be supported and integrated into health interventions, respecting cultural preferences and traditions.

### 4.2 Limitation of the study

The tendency of respondents to over report their dietary consumption couldn’t be ruled out, despite the use of strong measurement during data collection, which may subjected to social desirability. Additionally, since this is a cross-sectional study, it cannot establish causality. Furthermore, due to the multicultural and diverse nature of the community, minor food items consumed by the majority might not have been adequately captured.

## 5 Conclusion

Three distinct dietary patterns: namely “Cereals-Pulses and Dairy” pattern, “Nutrient-Dense” pattern and “Leafy local food” pattern were identified were identified. Factors associated with these distinct dietary patterns included place of residence, socioeconomic status, educational level, dietary knowledge, food aversion, number of pregnancies, and maternal decision-making autonomy. Also, under nutrition among pregnant women was found to be high and associated with the consumption of the “Nutrient-Dense” pattern and “Leafy Local Food” pattern. Therefore, concerned health authorities should strengthen dietary counseling during pregnancy, strengthen family planning services, and promote women’s education. Decision-makers should also focus on improving the socioeconomic status of households and women’s decision-making autonomy. Further studies are needed to address the nutrient and caffeine content of locally prepared coffee leaf tea beverage.

## Data Availability

The original contributions presented in the study are included in the article/Supplementary material, further inquiries can be directed to the corresponding author.
