# Supplementary material for: Factors associated with dietary patterns (DPS) and nutritional status among pregnant women in AM-HDSS, South Ethiopia
Source: Front Nutr. 2024 Sep 12;11:1443227. doi: 10.3389/fnut.2024.1443227 (PMC11428162; doi:10.3389/fnut.2024.1443227)
Supplement: Supplementary file 1 [file Table_1.DOCX]

Supplementary Material

Supplementary Table; Factors associated with identified dietary patterns of pregnant women Gamo Zone, South Ethiopia in bivariate ordinal logistic regression analysis

| **Variable** | **Category** | **“Cereals-Pulses and Dairy”** | | **“Nutrient-Dense”** | | **“Leafy local food”** | |
| --- | --- | --- | --- | --- | --- | --- | --- |
|  |  | **COR(95% CI)** | **P-value** | **COR(95% CI)** | **P-value** | **COR(95% CI)** | **P-value** |
| Age of the respondent | Less than 20 years | 1 |  | 1.67 (0.82, 3.36) | 0.15 | 1 |  |
|  | 20 to 35 years | 1.16 (0.75, 1.78) | 0.50 | 1.73 (0.95, 3.14) | 0.07 | 1.25 (0.82, 1.93) | 0.29 |
|  | 36 years and more | 1.28 (0.64, 2.56) | 0.48 | 1 |  | 1.65 (0.82, 3.32) | 0.15 |
| Place of residence | Rural | 1 |  | 1 |  | 1 |  |
|  | Urban | 2.45 (1.5, 3.96) | 0.01 | 1.87 (1.16, 3.02) | 0.01 | 1.16 (0.73, 1.86) | 0.51 |
| Religion | Orthodox | 1 |  | 1.09 (0.76, 1.57) | 0.61 | 1.01 (0 .70, 1.43) | 0.98 |
|  | Protestant | 1.07 (0.75, 1.53) | 0.69 | 1 |  | 1 |  |
| Educational status | No formal education | 1 |  | 1 |  | 2.20 (1.63, 2.98) | 0.01 |
|  | Formal education | 1.41 (1.05, 1.89) | 0.02 | 1.48 (1.10, 1.99) | 0.01 | 1 |  |
| Occupational status | House wife | 1 |  | 1 |  | 1 |  |
|  | Farmer | 0.86 (0.56, 1.30) | 0.46 | 0.71 (0.46, 1.10) | 0.12 | 1.64 (1.06, 2.56) | 0.02 |
|  | Merchant | 0.88 (0.56, 1.38) | 0.57 | 1.19 (0.75, 1.89) | 0.45 | 0.84 (.53, 1.33) | 0.45 |
|  | Government employ | 1.68 (0 .89, 3.17) | 0.10 | 2.77 (1.41, 5.43) | 0.03 | 0.98 (0.54, 1.81) | 0.95 |
|  | Student | 2.56 (1.03, 6.38) | 0.04 | 1.84 (0.81, 4.23) | 0.14 | 0.93 (0.41, 2.12) | 0.86 |
| Households decision-making | By both | 1.23 (0.85, 1.77) | 0.26 | 1.91 (1.32, 2.78) | 0.01 | 1.52 (1.06, 2.19) | 0.02 |
|  | Mainly by husband | 1 |  | 1 |  | 1 |  |
| Household family member | Less than five | 1 |  | 1 |  | 1.83 (1.37, 2.45) | 0.01 |
|  | Five and more | 1.26 (0 .95, 1.68) | 0.10 | 1.25 (.94, 1.66) | 0.13 | 1 |  |
| Household wealth status | Low | 1 |  | 1 |  | 1.49 (1.05, 2.11) | 0.02 |
|  | Middle | 1.93 (1.36, 2.75) | 0.01 | 1.24 (0.88, 1.76) | 0.21 | 1.33 (0.94, 1.90) | 0.12 |
|  | High | 2.50 (1.74, 3.59) | 0.01 | 1.47 (1.04, 2.08) | 0.03 | 1 |  |
| Nutritional knowledge | Poor knowledge | 1 |  | 1 |  | 1 |  |
|  | Moderate knowledge | 1.04 (0.76, 1.40) | 0.81 | 0.90 (0.67, 1.22) | 0.51 | 1.44 (1.06, 1.95) | 0.02 |
|  | Good knowledge | 1.47 (0.91, 2.38) | 0.11 | 1.43 (0.88, 2.31) | 0.15 | 1.74 (1.07, 2.83) | 0.02 |
| Number of pregnancy | Primigravida | 2.12 (1.34, 3.34) | 0.01 | 1.12 (0.72, 1.77) | 0.61 | 1 |  |
|  | Multigravida | 2.14 (1.45, 3.15) | 0.01 | 1.36 (0.93, 1.99) | 0.11 | 1.19 (0.84, 1.70) | 0.31 |
|  | Grand Multi | 1 |  | 1 |  | 1.28 (0.82, 2.01) | 0.27 |
| Number of delivery | Primipara | 1.04 (0.59, 1.82) | 0.89 | 2.25 (1.26, 4.02) | 0.01 | 1 |  |
|  | Multipara | 1.14 (0 .67, 1.93) | 0.63 | 1.99 (1.15, 3.46) | 0.01 | 1.17 (0.81, 1.68) | 0.39 |
|  | Grand Multipara | 1 |  | 1 |  | 1.31 (0.75, 2.31) | 0.33 |
| ANC visit | Yes | 1.01 (0.75, 1.37) | 0.94 | 1.33 (.98, 1.81) | 0.06 | 1 |  |
|  | No | 1 |  | 1 |  | 1.28 (0 .94, 1.74) | 0.11 |
| Had nausea/vomiting | Often | 1.31 (0.90, 1.90) | 0.16 | 1.11 (.77, 1.61) | 0.57 | 1 |  |
|  | Some times | 1.26 (0.91, 1.75) | 0.16 | 1.29 (0.93, 1.78) | 0.13 | 1.42 (0 .96, 2.11) | 0.08 |
|  | No | 1 |  | 1 |  | 1.84 (1.26, 2.67) | 0.01 |
| Food aversion | Yes | 1.06 (0.71, 1.57) | 0.29 | 1.83 (1.20, 2.79) | 0.01 | 1 |  |
|  | No | 1 |  | 1 |  | 1.18 (0 .78, 1.78) | 0.41 |
| Nutritional counseling | Yes | 1.16 (0.79, 1.70) | 0.80 | 1.17 (0.79, 1.71) | 0.42 | 1 |  |
|  | No | 1 |  | 1 |  | 1.67 (1.15, 2.45) | 0.01 |
